# Meristem culture and subsequent micropropagation of Chilean strawberry (*Fragaria chiloensis* (L.) Duch.)

**DOI:** 10.1186/s40659-017-0125-8

**Published:** 2017-06-02

**Authors:** Karla A. Quiroz, Miguel Berríos, Basilio Carrasco, Jorge B. Retamales, Peter D. S. Caligari, Rolando García-Gonzáles

**Affiliations:** 1 Instituto de Biología Vegetal y Biotecnología, Universidad de Talca, Avenida Lircay s/n., Talca, Chile; 2 Centro de Biotecnología de los Recursos Naturales (CENBio), Facultad de Ciencias Agrarias y Forestales, Universidad Católica del Maule, Avenida San Miguel, 3605 Talca, Chile; 3 Facultad de Agronomía e Ingeniería Forestal, Pontificia Universidad Católica de Chile, Vicuña Mackenna, Macul, 4860 Santiago, Chile; 4 Centro de Mejoramiento Genético y Fenómica Vegetal, Universidad de Talca, Avenida Lircay s/n., Talca, Chile; 5 Sociedad de Investigación y Servicios, BioTECNOS Ltda., Talca, Chile

**Keywords:** *Fragaria chiloensis*, Meristem culture, Plant growth regulators, Virus elimination, Plant morphogenesis

## Abstract

**Background:**

Vegetative propagation of *Fragaria* sp. is traditionally carried out using stolons. This system of propagation, in addition to being slow, can spread plant diseases, particularly serious being viral. In vitro culture of meristems and the establishment of micropropagation protocols are important tools for solving these problems. In recent years, considerable effort has been made to develop in vitro propagation of the commercial strawberry in order to produce virus-free plants of high quality. These previous results can serve as the basis for developing in vitro-based propagation technologies in the less studied species *Fragaria chiloensis*.

**Results:**

In this context, we studied the cultivation of meristems and establishment of a micropropagation protocol for *F. chiloensis*. The addition of polyvinylpyrrolidone (PVP) improved the meristem regeneration efficiency of *F. chiloensis* accessions. Similarly, the use of 6-benzylaminopurine (BAP) in the culture media increased the average rate of multiplication to 3–6 shoots per plant. In addition, the use of 6-benzylaminopurine (BAP), had low levels (near zero) of explant losses due to oxidation. However, plant height as well as number of leaves and roots were higher in media without growth regulators, with average values of 0.5 cm, 9 leaves and 4 roots per plant.

**Conclusions:**

For the first time in Chilean strawberry, meristem culture demonstrated to be an efficient tool for eliminating virus from infected plants, giving the possibility to produce disease free propagation material. Also, the addition of PVP into the basal MS medium improved the efficiency of plant recovery from isolated meristems. Farmers can now access to high quality plant material produced by biotech tools which will improve their technological practices.

**Electronic supplementary material:**

The online version of this article (doi:10.1186/s40659-017-0125-8) contains supplementary material, which is available to authorized users.

## Background

The Chilean strawberry (*Fragaria chiloensis* L. Duch.) is a berry fruit of great agricultural and commercial potential due to its excellent organoleptic properties, its exquisite aroma and flavour, and the exotic white/pink colour of its fruits [1]. This species is one of the progenitors of the cultivated strawberry (*Fragaria x ananassa Duch.*) and grows naturally in Chile, Hawaii, and the west coast of the United States [2]. This wild material has also attracted interest from cultivated strawberry breeding programs due to a number of interesting agronomic characteristics, such as: resistance to pests and diseases, tolerance to drought and salinity, and fruits with good organoleptic characteristics. In *F. chiloensis*, the current and traditional form of plant propagation is through stolons. This practice generates plants of poor quality [3] because such propagation often also transfers incipient diseases that reduce agricultural yields. This is especially important in relation to viruses that move through vascular plant tissues [4]. A biotechnological alternative to obtain large quantities of healthy plants is the isolation of meristematic tissue, since this is generally free of viruses because its active cell division reduces differentiation of vascular tissues [5]. Once isolated, this meristematic tissue can be cultivated; micropropagation protocols can be developed that enable an adequate supply of genetically homogeneous and disease-free plant material.

Meristems are the centres of plant growth located in apical and lateral buds as well as roots of berry species, especially in *Fragaria* sp. [6, 7]. Thus, meristematic tissue culture is an appealing technique to eliminate pathogenic bacteria, fungus, and viruses carried by adult plants. However, a number of constraints need to be overcome in order to facilitate meristem isolation and establishment in in vitro conditions [8] including: reducing the release of phenolic compounds from the tissues into the culture medium, and appropriate environmental conditions, such as suitable temperatures.

In *Fragaria* sp., Jadwiga et al. [9] found that plants derived from in vitro propagation behaved better under field conditions since they produced more leaves, stolons and flowers than those propagated by stolons. In addition, the in vitro raised plants were also more resistant to leaf burn induced by frost stress.

Subsequent, in vitro propagation of *Fragaria* sp. is effected by several factors that should be considered for establishing reliable micropropagation protocols. High concentration of mineral salts in the basal Murashige and Skoog culture medium (MS medium) promoted efficient organogenesis in three cultivars of *Fragaria* sp., which were characterized by lack of morphological development [10]. Similarly, it has been determined that the proliferation rate was genotype-dependent, while the physiological age of the explants cultured in vitro did not affect plant morphogenesis [10].

Jemmali et al. [11] found morphological and hormonal differences between in vitro plants of *Fragaria x ananassa*, regenerated from axillary and stipulary buds. They concluded that the adventitious shoots from bud stipules had a higher rate of multiplication and greater concentration of cytokinins, while buds formed from axillary buds had light green pigmentation in their leaves, which would indicate lower formation of chlorophyll.

Earlier, Bhatt and Dhar [12] introduced protocols to develop in vitro material of *F.* x *ananassa* from nodal segments. Similarly, Donnoli et al. [13] established a plant regeneration protocol for the wild species *Fragaria vesca* and for three cultivars (*Clea, Irving and Paros*) of *Fragaria x ananassa.* In relation to *F. chiloensis,* Paredes and Lavin [14] established a protocol of introduction of meristematic buds from stolons using a basal MS medium with the addition of indolebutyric acid, 6-benzylaminopurine and GA_3_. Explants were cultured at 25 °C, then, differentiation and proliferation of induced buds took place in a different basal culture of MS medium supplemented with different levels of the same compounds. Finally, the buds were elongated and rooted in MS medium with only 6-benzylaminopurine.

Against this background, this paper seeks to investigate responses to culture and media conditions to optimise an efficient and reliable protocol for in vitro establishment of meristems and subsequent micropropagation of *F. chiloensis* in order to produce virus free and genetically homogeneous plants using as models two selected accessions of this species.

## Results

### Morphogenic response of isolated meristems

**Fig. 1:**
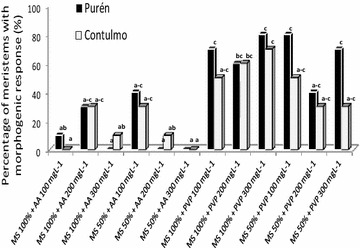
Effect of media dilution (MS 100% and MS 50%) and antioxidants on *Fragaria chiloensis* meristems. Purén and Contulmo represents the *Fragaria chiloensis* accessions. *PVP* polyvinylpyrrolidone, *AA* ascorbic acid. Analysis done with Kruskal–Wallis. Treatments with common letters are not significantly different (P < 0.05). Evaluation was done 6 weeks after culture

As shown in Fig. 1, the use of PVP in the culture medium increased the percentage of meristems with morphogenic response, in relation to the use of ascorbic acid. However, there were no significant differences in effects between the different concentrations of PVP used. There were also no significant differences detected in relation to the medium strength (concentration of salts in MS medium).

### Effects of plant growth regulators on morphogenesis of meristem-derived plantlets

#### Auxin/cytokinin interactions

**Fig. 2:**
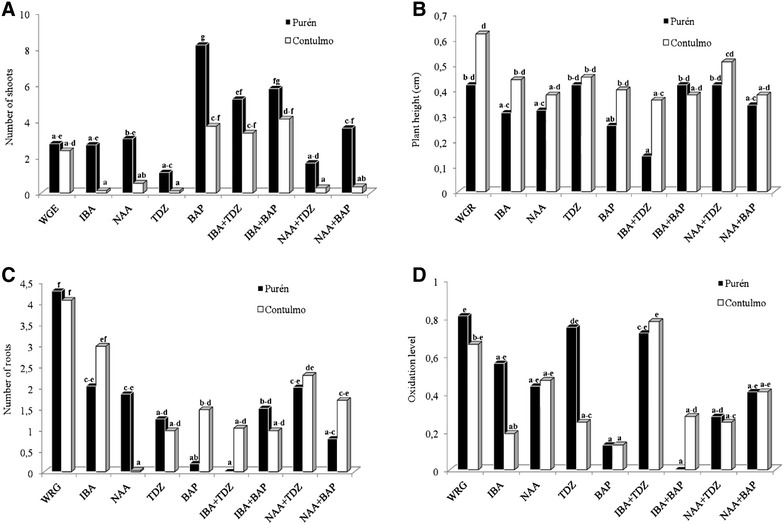
Effect of auxins (NAA, IBA) and cytokinins (TDZ, BAP) on plant morphogenesis of *Fragaria chiloensis.* **A** number of shoots; **B** plant height; **C** number of roots; and oxidation level (**D**). Purén and Contulmo represent the *Fragaria chiloensis* accessions. *WGR* without growth regulator. Treatments with *common letters* are not significantly different (P < 0.05). Weighted oxidation level ranged from 0 (for no oxidation) up to 4 (for 76–100% oxidized). Evaluation was done 6 weeks after culture

The analyses of results are shown in Table 1 In which it can be seen that the number of shoots and leaves were strongly influenced by the addition of auxins and cytokinins as well as the interaction between these growth regulators. Plant height was significantly influenced by the accession but no other effects were detected. In relation to the number of roots, there was a significant effect of cytokinin in its own right and, as an interaction with auxin, although auxins showed no significant effect itself. The more detailed effects can be seen in Fig. 2, where Fig. 2A shows that the cytokinin BAP, by itself, was effective in generating high number of shoots giving values of 8.2 and 3.7 shoots per explant, for Contulmo and Purén, respectively. The auxin, IBA was also effective when used in combination with either of the cytokinins. However, number of leaves, plant height (Fig. 2B), and number of roots (Fig. 2C) were not influenced by the use of auxins or cytokinins. The best performance was shown in media without growth regulators (WGR), which had 9–10 leaves, reached heights of between 0.4 and 0.6 cm, and had 4 or more roots per plant.

**Table 1:** Analysis of the effects and interactions of accession (Purén or Contulmo), level of auxin (NAA or IBA) and level of cytokinin (TDZ or BAP) on: number of shoots, number of leaves, plant height and number of roots in plants of *Fragaria chiloensis* propagated for 6 weeks

	Number of shoots			Number of leaves		Plant height		Number of roots	
	df	MS	P	MS	P	MS	P	MS	P
Accession	1	161.50	**	0.96	ns	0.33	*	1.13	ns
Auxin	2	49.77	**	80.31	**	0.09	ns	5.95	ns
Cytokinin	2	89.10	**	46.54	**	0.03	ns	29.86	**
Interactions									
Accession * auxin	2	0.56	ns	7.59	ns	0.01	ns	1.48	ns
Accession * cytokinin	2	9.93	ns	12.68	ns	0.02	ns	2.77	ns
Auxin * cytokinin	4	43.94	**	54.41	**	0.14	ns	23.81	**
Accession * auxin * cytokinin	4	7.09	ns	13.42	ns	0.04	ns	5.77	*
Error	126	7.53		8.83		0.06		2.03	
Total	143								

*df* degrees of freedom, *MS* mean squares, *ns* not significant

* (P < 0.05) significant difference

** (P < 0.01) highly significant difference

In terms of oxidation (Fig. 2D), the results were less clear but interestingly showed that the use of BAP (and for Purén when in combination with IBA) produced low levels of oxidation, resulting in healthier plants.

#### Cytokinins/gibberellic acid (GA_3_) interactions

**Fig. 3:**
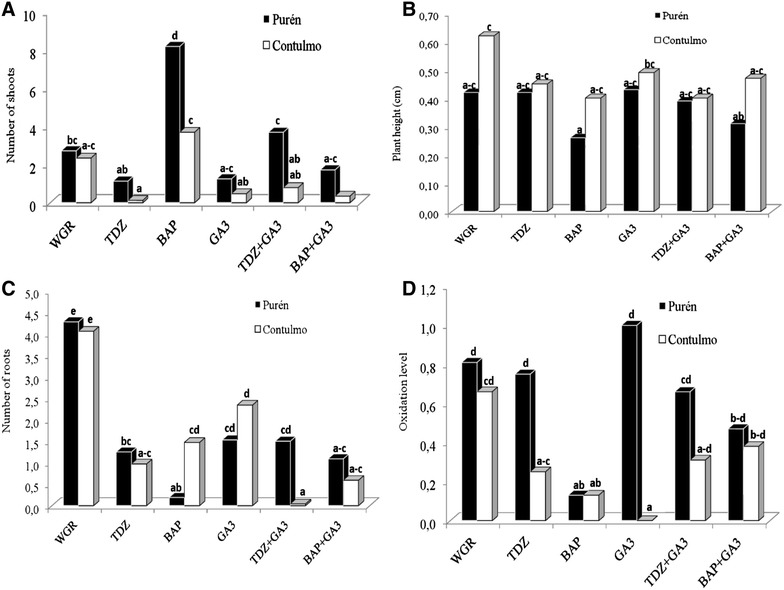
Effect of cytokinins (TDZ, BAP) combined with GA_3_ on plant morphogenesis of *Fragaria chiloensis.* **A** number of shoots; **B** plant height; **C** number of roots; **D** and oxidation level (D). Purén and Contulmo represent the *Fragaria chiloensis* accessions. *WGR* without growth regulator. Treatments with *common letters* are not significantly different (P < 0.05). Weighted oxidation level ranged from 0 (for no oxidation) up to 4 (for 76–100% oxidized). Evaluation was done 6 weeks after culture

The analyses of the results are given in Table 2, while the responses to all the treatments are shown in Fig. 3. The analysis of plant height shows it was not significantly affected by the presence of cytokinin or GA_3_, while the interaction between cytokinins and GA_3_ did not has any influence neither. The number of leaves only showed a significant response to cytokinins according to the analysis in Table 2. The analysis of root number showed inhibitory effects of cytokinins or GA_3_ when they were added individually or combining each cytokinin with GA_3_ Thus the plants that grew without cytokinins or GA_3_ (WGR) had greater number of roots per plant (Fig. 3B, C).

**Table 2:** Analysis of the effects and interactions of accession (Purén or Contulmo), level of cytokinins (TDZ or BAP) and level of gibberellic acid (GA_3_) on: number of shoots, number of leaves, plant height and number of roots, in *Fragaria chiloensis* propagated for 6 weeks

	Number of shoots			Number of leaves		Plant height		Number of roots	
	df	MS	P	MS	P	MS	P	MS	P
Accession	1	79.60	**	0.27	ns	0.24	*	0.09	ns
Cytokinin	2	39.90	**	95.07	**	0.14	ns	50.16	**
GA_3_	1	65.70	**	33.74	ns	0.00	ns	17.44	**
Interactions									
Accession * cytokinin	2	11.23	ns	2.41	ns	0.04	ns	3.94	*
Accession * GA_3_	1	0.45	ns	7.36	ns	0.01	ns	2.48	ns
Cytokinin * GA_3_	2	85.04	**	33.93	ns	0.03	ns	11.71	**
Accession * cytokinin * GA_3_	2	13.30	ns	5.92	ns	0.01	ns	4.38	*
Error	84	6.39		11.15		0.05		1.16	
Total	95								

*df* degrees of freedom, *MS* mean squares, *ns* not significant

* (P < 0.05) significant difference

** (P < 0.01) highly significant difference

As can be observed in Fig. 3D, the lowest levels of explant oxidation were obtained in basal media supplemented with BAP. The effect of GA_3_ alone on oxidation was highly accession dependent, with high levels for the Purén plants, while the Contulmo plants showed values close to zero.

#### Auxins/gibberellic acid (GA_3_) interactions

**Fig. 4:**
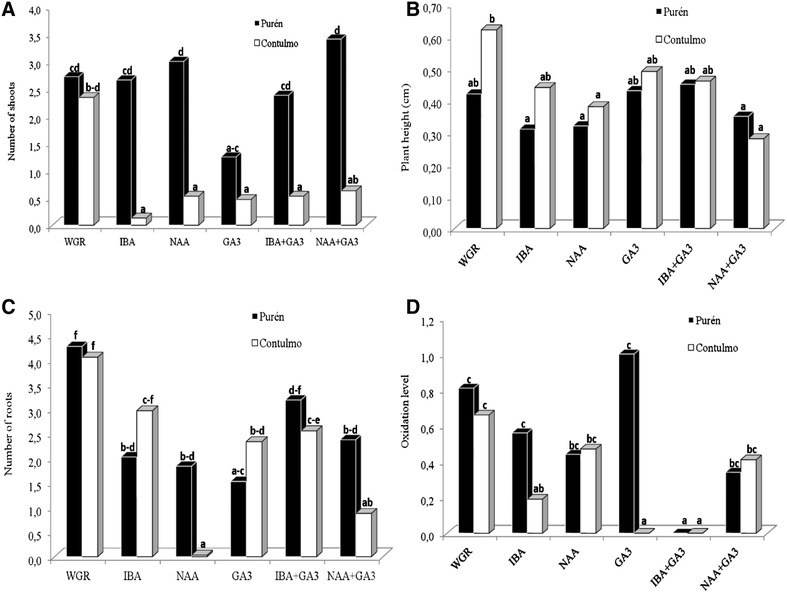
Effect of auxins (IBA, NAA) combined with GA_3_ on plant morphogenesis of *Fragaria chiloensis.* **A** number of shoots; **B** plant height; **C** number of roots; **D** and oxidation level (**D**). Purén and Contulmo represent the *Fragaria chiloensis* accessions. WGR = without growth regulator. Treatments with* common letters* are not significantly different (P < 0.05). Weighted oxidation level ranged from 0 (for no oxidation) up to 4 (for 76–100% oxidized). Evaluation was done 6 weeks after culture

Auxin on its own, when added to the basal medium, significantly affected the number of leaves, plant height and number of roots (Table 3). The number of leaves and roots were also affected by the interaction between auxins and GA_3_. Neither auxins nor GA_3_ influenced shoot formation. However, it is clear from Fig. 4A–D that the use of GA_3_ or auxins, either separately or together, did not generate more favourable responses than the control treatment (WGR). The oxidation levels (Fig. 4D) were lowest in media supplemented with IBA and particularly in combination with GA_3_.

**Table 3:** Analysis of the effects and interactions of: accession (Purén or Contulmo), level of auxin (IBA or NAA) and level of gibberellic acid (GA_3_) on: number of shoots, number of leaves, plant height and number of roots in *Fragaria chiloensis* propagated for 6 weeks

	Number of shoots			Number of leaves		Plant height		Number of roots	
	df	MS	P	MS	P	MS	P	MS	P
Accession	1	77.49	**	13.13	ns	0.10	ns	3.86	ns
Auxin	2	1.77	ns	105.18	**	0.20	*	28.04	**
GA_3_	1	4.93	ns	0.67	ns	0.00	ns	3.66	ns
Interactions									
Accession * auxin	2	9.29	ns	2.18	ns	0.04	ns	9.49	*
Accession * GA_3_	1	0.00	ns	0.51	ns	0.10	ns	0.03	ns
Auxins * GA_3_	2	8.98	ns	61.99	**	0.04	ns	20.59	**
Accession * auxin * GA_3_	2	0.74	ns	17.33	ns	0.00	ns	3.59	ns
Error	84	3.09		10.09		0.05		2.34	
Total	95								

*df* degrees of freedom, *MS* mean squares, *ns* not significant

* (P < 0.05) significant difference

** (P < 0.01) highly significant difference

#### Analyses of virus infection

The results of the virus analyses carried out by the Agricultural and Livestock Service (Servicio Agrícola y Ganadero, SAG) on the donor plants are given in Table 4 and show that in both accessions, all donor plants analysed were infected by both SMYEV and SMoV. Analyses of the plants regenerated in vitro from the meristems introduced from the accession Contulmo were 100% free of SMYEV and SMoV viruses. In the case of the accession Purén, the effectiveness of the meristem culture was 78% effective in eliminating the SMYEV virus and 100% for the SMoV virus.

**Table 4:** Effectiveness of meristem culture for virus elimination in *Fragaria chiloensis* for two common and widely distributed viruses in Chile

Accession	Strawberry mild yellow edge virus				Strawberry mottle virus			
	Donor plants^a^		Isolated meristems		Donor plants^a^		Isolated meristems	
	Total^a^/analysed explants	Virus free (%)	Total/sampled	Virus free (%)	Total^a^/analysed explants	Virus free (%)	Total/sampled	Virus free (%)
Contulmo	3/20	0	360/20	100	3/20	0	360/20	100
Purén	3/20	0	360/20	78	3/20	0	360/20	100

^a^ Corresponds to the number of original plants per accession and the number of plants propagated from them and used as donors

## Discussion

As has been observed in *F. x ananassa* [6], the oxidation of meristems during the in vitro establishment process is a major problem affecting the development of virus free in vitro plantlets. Our results show that the addition of ascorbic acid did not reduce tissue oxidation or improved the morphogenic response in either accession of Chilean strawberry. This is in contrast to the results found for tomato by Bhatia and Ashwath [15]. On the other hand, the results clearly showed that PVP improved the morphogenic capacity of meristems, which coincides with results for chestnut trees [16] and *Aloe vera* [17].

Phenolic oxidation is also a problem prevalent in the growth and propagation of established in vitro plants and has both an environmental and genetic component. It has previously been established that oxidation of “in vitro established” explants can be controlled by modifying the environmental conditions of cultivation and the management of explants [18], or through the addition of antioxidants to the nutrient medium [15]. Here we have shown that BAP in the media reduces oxidation in *F. chiloensis* while GA_3_ had a particularly detrimental effect on one of the accessions.

The addition of cytokinins in the rooting phase had similar effect for both accessions of *F. chiloensis*, coinciding with the results reported for *F. x ananassa*, in that efficient rooting was obtained in all the genotypes evaluated when cytokinin was excluded from the culture medium [19]. However, interestingly, for both studied accessions of *F. chiloensis* included in the study, the addition of auxins did not significantly improve the formation of roots as compared to a hormone-free medium. This is in contrast with reports from the commercial strawberry (*F. x ananassa*), where root formation was only induced by the auxin IAA [20]. It may be relevant to note that auxins such as 2,4-D can also induce calli formation affecting the efficiency of root induction in commercial strawberry explants [21]. Also, it has been found that higher concentrations of auxins reduced root formation by an inhibitory effect of the produced calli [22].

Although further studies are desirable, for example, to discover the levels of endogenous hormones in this species, it appears that *F. chiloensis* produces sufficient endogenous auxin levels to induce rooting, and that the incorporation of additional amounts lessens this process, as previously documented for sweet potato [5] and henequen [23].

**Fig. 5:**
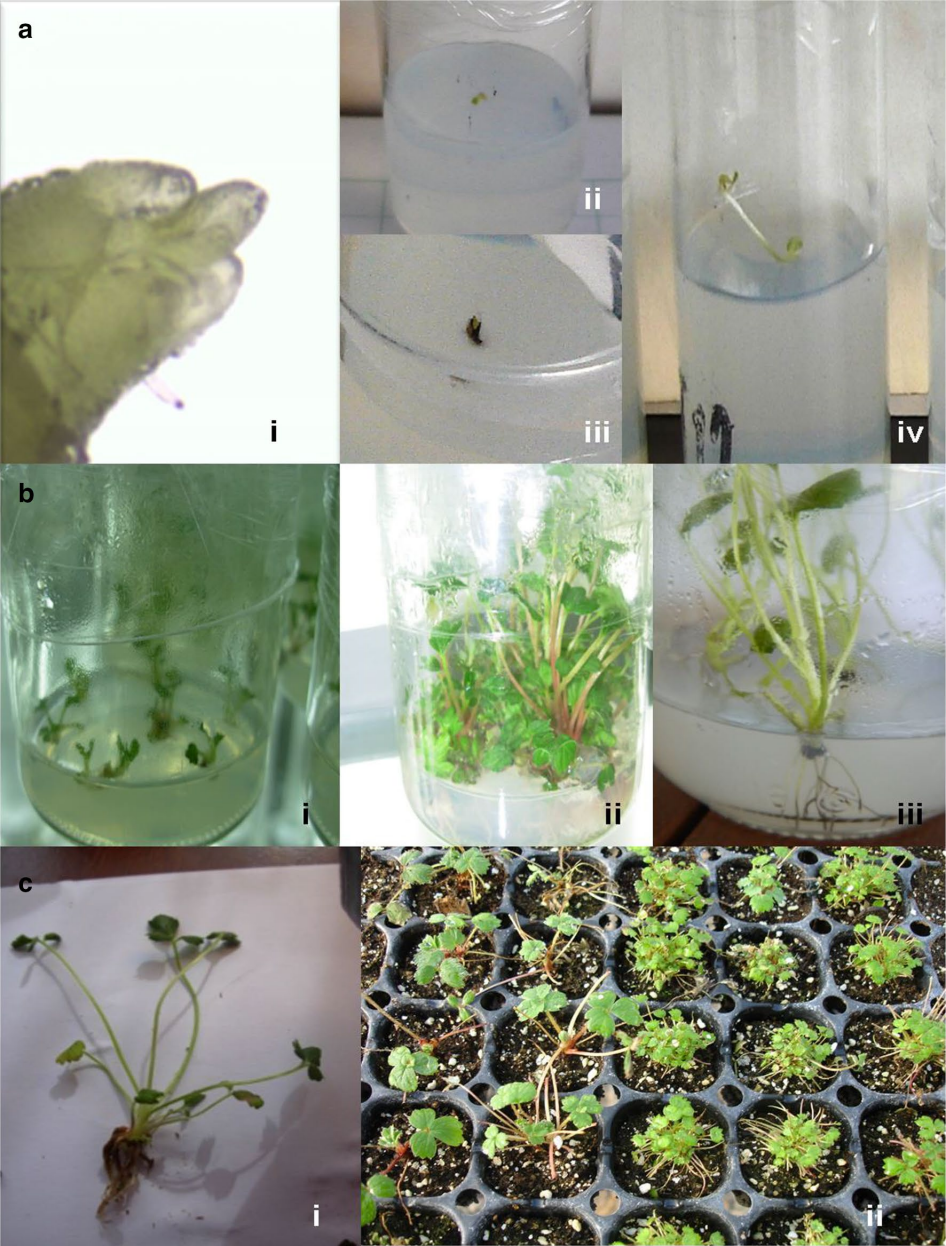
Different steps during meristem isolation and in vitro propagation of *Fragaria chiloensis.* **a** Meristem isolation and culture: *i* isolated meristem near to be cultivated, *ii* green meristem showing viability after culture, *iii* dead meristem after oxidation, *iv* plantlet shooting from an isolated meristem. **b** Morphogenic development of in vitro plantlets: *i* shoot induction from cultivated meristems, *ii* in vitro multiplication by adventitious shoot formation, *iii* rooting of in vitro plants. **c** Ex vitro adaptation of micropropagated plants: *i* a 35 days old plantlet ready for the ex vitro step; *ii* ex vitro plantlets 21 days after adaptation

The plants produced in this study developed into normal plants and were adapted successfully to ex vitro conditions. In these conditions the plants also grew normally and were morphologically similar to plants propagated by stolons (Fig. 5).

## Conclusions

According to these results, PVP improved the establishment of *F. chiloensis* meristems in culture by reducing oxidation levels, and as the tissue responses showed no significant differences between the concentrations tested; it appears that the lowest concentration (100 mg l^−1^) of this antioxidant should be added to the culture medium, for improving morphogenic responses and differentiation of whole plants.

The addition of BAP (0.5 mg l^−1^) in the culture media improved the subsequent in vitro multiplication, while also showing low levels of phenolic oxidation, suggesting that the use of this growth regulator is a suitable media component in the multiplication of this species.

Considering that the basal medium without plant regulators was more effective in inducing plant height, leaves and roots than those media supplemented with PGR’s, it would be appropriate to use this simple medium during the rooting or pre-acclimatisation step thus avoiding unnecessary stages of transfer to new media and helping make the micropropagation process more cost-effective. It is important to point out that from the results of direct virus testing, the meristem culture carried out on *F. chiloensis* was demonstrated to be effective in virus elimination.

## Methods

### Plant material and meristem isolation

This research used plants of the Chilean strawberry, *F. chiloensis* forma *chiloensis*, accessions Contulmo and Purén, kept in the genebank of the Experimental Station “Panguilemo” at the University of Talca, located at 35° 21′ latitude south, 111 meters above sea level. For the establishment of the in vitro meristems, stolons were collected from ten donor plants and washed with running tap water. Subsequently, the stolons were soaked with agitation (at 40 rpm) in sterile distilled water for 5 min. Following this, the stolons were washed in a Tween 20 surfactant solution (0.1%) for 5 min, and then subjected to three washes with sterile water. Then, the stolons were immersed for 10 s in 70% ethanol and washed three times in sterile water. They were then disinfected for 10 min in a solution of sodium hypochlorite at 1.5% with 0.1% Tween 20. After three rinses with sterile water, the meristems were dissected under a stereoscope (20× or 40×, Olympus). The isolated meristems were inoculated onto semisolid MS medium.

To determine the effectiveness of meristem culture in relation to the elimination of two strawberry viruses [Strawberry Mild Yellow Edge Virus (SMYEV) and Strawberry Mottle Virus (SMoV)] samples of leaves of the donor plants from the genebank and from plants raised through meristem culture were sent to the Virology Laboratory of the Agricultural and Livestock Service of Chile (Servicio Agrícola y Ganadero, SAG) for virus testing. Certification of virus free plants was carried out according to standard protocols for these viruses [24, 25]. To test virus presence from donor plants in the bank, 20 propagated plants used as meristem donors and previously propagated from the original accessions were analysed. In the case of the isolated meristems, twenty plants were sampled from 360 isolation events to detect each virus.

### Effect of the basal medium and antioxidants on meristem survival after isolation and disinfection

The effect of culture medium on meristem oxidation was evaluated by using MS [26] basal medium (salts and vitamins) without dilution and MS basal medium diluted to 50%. Furthermore, the addition of different concentrations of the antioxidants PVP (Duchefa Biochemie, GrupoBios, Haarlem, Netherlands) and ascorbic acid (Merck, Darmstadt, Germany) each at concentrations of 100, 200 and 300 mg l^−1^, were evaluated. Disinfected meristems were cultivated to 20–21 °C and maintained in complete darkness until the early signs of morphogenic activity (shooting, leaf development, rooting or calli production) were observed.

To investigate this, 12 treatments for each accession were generated, each with 10 meristem introduction events. To interpret the results and identify the best treatment for meristem survival after disinfection and in vitro establishment, a Kruskal–Wallis test was performed (P ≤ 0.05).

### Effects of plant growth regulators on the in vitro propagation

#### Plant material and general conditions

Once established, the plants differentiated from meristems were cultivated in 200 ml glass vessels containing 25 ml of solid MS medium [26] supplemented with 3% sucrose, 7.5 g l^−1^ of agar agar (TCL, Santiago, Chile) and pH 5.7–5.8, adjusted before sterilization by autoclaving (Huxley, HL-341, Taipei, Taiwan) for 20 min at 121 °C and 1 kg cm^−2^ of pressure. All experiments were carried out with plants grown for four weeks at 24 ± 2 °C under a photoperiod of 16 h light using fluorescent white light (40 Watt tubes, Philips, Holland) generating a light intensity of 60 μmol m^−2^ s^−1^.

Four stem segments, each containing two buds, with 1 cm of petiole, without leaves and roots, were placed in each vial containing 25 ml of semi-solid regeneration media. Explants were cultivated in their respective treatments for 6 weeks at a temperature of 24 ± 2 °C and a photoperiod of 16 h light (60 µmol m^−2^ s^−1^). Each treatment was replicated eight times.

### Effects of plant growth regulator interactions on plant morphogenic response

#### Auxins/cytokinins interactions

The interactions between the auxins: indolebutyric acid (IBA) (Duchefa Biochemie, GrupoBios, Haarlem, Netherlands) and naphthaleneacetic acid (NAA) (Duchefa Biochemie), and the cytokinins: thidiazuron (TDZ) (Duchefa Biochemie) and 6-benzylaminopurine (6-BAP) (Phytotechnology Laboratories, Genexpress, Philekorea, South Korea) on Chilean strawberry morphogenic response were studied. All growth regulators were added to the medium before sterilization by autoclaving (121 °C and 1 kg cm^−2^ pressure for 20 min). Concentration of auxins in the medium was set at 0.3 mg l^−1^ and in the case of cytokinins was 0.5 mg l^−1^. The effect of no plant growth regulator addition as well as the isolated effect of each plant growth regulator was also evaluated. In total, 9 treatments were assessed for each of the two Chilean strawberry accessions.

#### Cytokinins/gibberellic acid (GA_3_) interactions

The interaction of cytokinins and GA_3_ was investigated in terms of the induction of plant morphogenesis in *F. chiloensis* by combining 0.5 mg l^−1^ of TDZ or BAP with 1 mg l^−1^of GA_3_ (Duchefa Biochemie). In this trial, the effect of the basal medium without plant growth regulator was also evaluated as well as the isolated effect of each plant growth regulator, giving six treatments in total for each of the two accessions.

#### Auxins/gibberellic acid (GA_3_) interactions

The effects on plant morphogenesis of the interactions between the auxins NAA and IBA and gibberellin, GA_3_, were evaluated. Auxins were added as 0.3 mg l^−1^ while GA_3_ was added as 1 mg l^−1^. As in the above-mentioned experiments, the isolated effect of auxins and GA_3_ in the medium as well as no addition of plant growth regulator was evaluated. For each accession, this meant six treatments.

### Design and statistical analysis

The statistical design had a multifactorial structure with three factors for each experiment. The homogeneity of variances was determined by Levene’s test (ά = 0.05). To compare and then to choose the best treatments, the multiple range LSD test (P < 0.05) (Least Significant Difference) was used. Qualitative parameters were analysed using non-parametric statistics with Kruskal–Wallis test (P < 0.05). All statistical analyses were performed with the software InfoStat versión 2012 (Grupo InfoStat, FCA, Universidad Nacional de Córdoba, Argentina).

In order to choose the best regeneration medium, number of shoots and roots per explant, plant height and number of leaves on the regenerated plants, as well as calli production, were evaluated. Similarly, the effect of each treatment on the physiological quality of shoots produced was evaluated by measuring plant survival and explant oxidation. To calculate the degree of oxidation, first an arbitrary visual scale was developed by considering the expression of the phenolization of the explants. The scale was set as follows: Value 0, if no oxidation was observed; Value 1, if explant oxidation or death was between 1 and 25% of the explant area; Value 2, if explant oxidation or death was between 26 and 50% of the explant area; Value 3, if explant oxidation or death was between 51 and 75% of the explant area; Value 4, if explant oxidation or death was between 76 and 100% of the explant area.

Then the weighted degree of oxidation, which used the visual scores but considered the frequency and intensity of explant damage, was calculated as follows [27]:

$${\text{P}} = \left[ {\sum {({\text{n}}*{\text{v}})/{\text{CM}}*{\text{N}}} } \right]*100$$

where P = weighted degree of severity of oxidation; n = number of explants of each class of the scale; v = numerical value of each class; CM = higher value of the scale; N = total number of explants in the sample.

## Additional file

**Additional file 1.** Raw data.

## Abbreviations

PVP: polyvinylpyrrolidone
AA: ascorbic acid
BAP: 6-benzylaminopurine
IBA: indolebutyric acid
TDZ: thidiazuron
NAA: naphthaleneacetic acid
GA_3_: gibberellic acid
SMYEV: strawberry mild yellow edge virus
SMoV: strawberry mottle virus
SAG: Servicio Agrícola y Ganadero
